# Highly expressed of SERPINA3 indicated poor prognosis and involved in immune suppression in glioma

**DOI:** 10.1002/iid3.515

**Published:** 2021-08-27

**Authors:** Qing Yuan, Song‐Quan Wang, Guang‐Tao Zhang, Jie He, Zhi‐Dan Liu, Ming‐Rong Wang, Hong‐Qing Cai, Jing‐Hai Wan

**Affiliations:** ^1^ Department of Neurosurgery, National Cancer Center/National Clinical Research Center for Cancer/Cancer Hospital Chinese Academy of Medical Sciences and Peking Union Medical College Beijing China; ^2^ State Key Laboratory of Molecular Oncology, National Cancer Center/Cancer Hospital Chinese Academy of Medical Sciences and Peking Union Medical College Beijing China

**Keywords:** glioma, immune response, prognosis, SERPINA3

## Abstract

**Introduction:**

The prognosis of patients with glioma is dismal. It has been reported that Serpin peptidase inhibitor clade A member 3 (SERPINA3) is associated with the mobility and invasion of tumor cells. Our study was designed to explore the value of *SERPINA3* messenger RNA (mRNA) expression in the biological process, prognosis, and immune significance in glioma.

**Methods:**

We analyzed the biological functions of SERPINA3 through data from the Chinese Glioma Genome Atlas databases. Differentially expressed genes and enrichment analysis were performed and correlations between *SERPINA3* expression and immune cell infiltration were analyzed. Further, we validated the expression and the survival prediction role of SERPINA3 by using tissue microarrays and RNAscope in situ hybridization in 321 gliomas. The correlations between the expression and clinical‐pathological parameters as well as other biomarkers were examined.

**Results:**

Univariate and multivariate regression both indicated that the level of *SERPINA3* transcript represented an independent prognostic factor. High levels of *SERPINA3* correlated with poor survival in patients with glioma. Expression of *SERPINA3* mRNA was observed positively correlated with MCM6, *IGFBP2*, and FKBP10. Enrichment analysis showed SERPINA3 mainly enriched in immune‐related terms and signaling pathways including MAPK, TNF, P53, PI3K‐Akt, nuclear factor‐κB. Immune infiltration analysis further declare the *SERPINA3* expression negatively correlated with levels of Macrophages M1, native CD4^+^ T cell, monocytes, and Mast cell activated. And overexpression of *SERPINA3* correlated with low CD4^+^ T cell infiltration in glioma tissues.

**Conclusions:**

SERPINA3 may play a key role in the biological process of glioma cells especially in immune suppression activities. SERPINA3 may serve as an independent survival prediction factor in glioma patients.

## Graphical abstract

We observed that serpin peptidase inhibitor clade A member 3 (SERPINA3) messenger RNA was overexpressed in glioma tissues and involved in proliferation procession of glioma cells. SERPINA3 participate in promoting immune suppression in glioma TME. SERPINA3 could serve as a novel prognosis biomarker and therapy target in glioma.

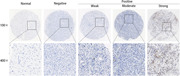

## 1 INTRODUCTION

Gliomas are the most prevalent primary tumours of the central nervous system, representing approximately 80% of malignant brain tumours^1^ and are responsible most deaths associated to primary brain tumours. Although concurrent radio‐ and chemoradiotherapy followed by chemotherapy after surgical removal of the tumour has become the standard treatment for patients with glioma, patient survival is still unsatisfactory. In particular, patients with high‐grade glioma (World Health Organization grade IV) have a median overall survival (OS) of 15–17 months upon clinical management.^2^, ^3^, ^4^ In recent years, research has focused on aberrant molecular alterations in gliomas. *IDH1/2* mutation,^5^ 1p/19q co‐deletion,^6^ *TERT* promoter mutation^7^ and several other markers have been used to define subtypes of gliomas. However, there are few therapeutic targets with potential clinical applications. Thus, more studies are needed to explore the specific molecular aberrations and propose new therapeutic regimens to improve the prognosis of patients.

Serpin peptidase inhibitor clade A member 3 (SERPINA3), also named alpha‐1‐antichymotrypsin, is a 55–66 kDa secreted serine protease that inhibits the activity of several serine proteases.^8^ Previous studies have shown that SERPINA3 plays an important role in cytokinesis, proliferation, apoptosis, and tumour metastasis.^9^, ^10^ Furthermore, SERPINA3 was reported to participate in multiple immunisation activities^10^ and that may serve as a potential immune therapy target in endometrial carcinoma.^11^ Nimbalkar et al.^12^ found that SERPINA3 contributes to proliferation, invasion, migration, and transition to mesenchymal phenotypes of glioma cells. Moreover, Luo et al.^13^ demonstrated that high levels of *SERPINA3* messenger RNA (mRNA) are correlated with poor prognosis in patients with glioma through real‐time polymerase chain reaction. However, SERPINA3 expression in glioma specimens detected by in situ methods has not been previously investigated, and the biological function of SERPINA3 in glioma has not been systematically analysed.

In the present study, the expression of *SERPINA3* mRNA was first detected in 321 glioma and 13 normal tissues by RNA in situ hybridization. The relationship between *SERPINA3* levels and the survival, clinical parameters, and expression of other biomarkers of glioma patients was analysed. Data from the Chinese Glioma Genome Atlas (CGGA) database were included as a test group. Furthermore, the biological function of SERPINA3 in glioma cells was explored. This study is expected to provide valuable information on the role of SERPINA3 as a novel biomarker and potential target in glioma.

## 2 MATERIALS AND METHODS

### 2.1 Data mining from public databases

Gene expression data and corresponding clinical information from the CGGA were downloaded (LGG + GBM) (http://www.cgga.org.cn/, DataSet ID: mRNAseq_693 and mRNAseq_325, Data type: RNA sequencing). Then we loaded these two sets of data into limma and sva packages in R software (R version 4.1.0: http://www.r-project.org/) to integrate and correction of gene expression data from glioma samples.

### 2.2 Differentially expressed genes (DEGs) and enrichment analysis

By analysis of gene expression data of CGGA, the sample of 1018 glioma patients were divided into two subgroups, according to the optimal cut‐off point of *SERPINA3* mRNA expression, *SERPINA3* ^high^ VS *SERPINA3* ^low^. Gene expression differences between *SERPINA3* ^high^ and *SERPINA3* ^low^ were identified within DESeq. 2. DEGs were determined by Wilcoxon rank‐sum test with *q* = 0.05 and fold change >1 after log2 transformation as the significance threshold and was used to create a volcano map. The genes with |log2Foldchange| >1.5 and adjusted *p* value of <.05 were regarded as the DEGs in the two groups. Gene Ontology (GO) functional analysis and Kyoto Encyclopedia of Genes and Genomes (KEGG) on the DEGs between groups of *SERPINA3* ^high^ & *SERPINA3* ^low^ were performed by the clusterProfiler package. Adjusted *p* value of <.05 were seen as significant.

### 2.3 Analysis of immune cell infiltration

Correlations between tumor‐immune infiltrating cells (TIICs) and the *SERPINA3* expression were analyzed. The proportions of the 21 TIICs from each sample were determined by using the “CIBERSORT” (R package). Then the level correlation of any two of the 21 TIICs was cauculated through spearman test. Finally, for each of TIICs, the difference of immune cells containment was analyzed between subgroups of *SERPINA3* ^high^ and *SERPINA3* ^low^.

To futher test the correlation of *SERPINA3* expression with the immune inflammation of glioma, we analysis the immune inflammation through TIMER, CIBERSORT, CIBERSORT‐ABS, QUANTI‐SEQ, XCELL, and EPIC methods^14^ in glioma patients from The Cancer Genome Atlas (TCGA). Then correlation analysis between *SERPINA3* mRNA expression and levels of immune cells were preformed through T test.

### 2.4 Tissue microarray (TMA) and in situ detection

A total of 321 gliomas and 13 paired normal or edematous brain tissue specimens were procured from the Department of Neurosurgery at the National Cancer Center/Cancer Hospital of Chinese Academy of Medical Sciences (CAMS). This research has been performed in accordance with the World Medical Association Declaration of Helsinki while approved by the Ethics Committee of CAMS (Number NCC2014G‐12). The collection and preparation of glioma TMAs were performed as we previously done.^15^ RNAscope in situ hybridization (RISH) and immunohistochemistry (IHC) were used for in situ detection in glioma TMAs as previously described.^15^, ^16^ Following probes and antibodies were used in this study: SERPINA3 (NM_001085.4, bp 353–1345 ACD#412671); IGFBP2 (NM_000597.2, bp 490–1423, ACD#313061), while Ubiquitin C (UBC, NM_021009, bp 342–1503, ACD#310041) and DapB (ACD#310043) were used as positive and negative control. Anti‐IDH1^R132H^ antibody (working solution, ZM0447; ZSGB‐BIO), anti‐MCM6 antibody (1:100, 13347‐2‐AP; Proteintech), anti‐FKBP10 antibody (1:2000, 50353; Sigma), and anti‐CD4 antibody (working solution, ZA‐0519; ZSGB‐BIO) were used in IHC assay. The evaluation of in situ signal scores were performed as before.

### 2.5 Survival analysis

Survival and survminer packages were loaded in R. Surv_cutpoint function was used to determine the optimal cut‐off point of *SERPINA3* mRNA expression in the datasets of CAMS & CGGA, and therefore divided the samples into high and low *SERPINA3* expression groups. OS curves were plotted according to the Kaplan–Meier method, with the log‐rank test applied for comparison. Moreover, univariate along with multivariate Cox regression models were constructed to explore the role of *SERPINA3* expression in survival of patients with glioma. Then time dependent receiver operator characteristics (ROC) curve analysis through survivalROC package in R was performed, at a threshold of area under curve (AUC) 0.7. Finally, a nomogram model including *SERPINA3* mRNA level was established through rms package in R to predict the survival prognosis of glioma patients in 1, 2, and 3 years after the operation.

### 2.6 Statistical analysis

The wilcoxon rank‐sum test was used to assess relations between the expression level of *SERPINA3* and corresponding clinical information. Significant differences between two groups were determined by the Mann–Whitney *U* test. The *χ* ^2^ test was used to assess the relationship between molecular alterations and clinico‐pathological parameters. A *p* value of <.05 was considered statistically significant. All tests were two‐sided.

## 3 RESULTS

### 3.1 Basic information of patients involved in the analysis

A total of 1339 patients with confirmed glioma (CAMS: 321 gliomas; CGGA:1018 gliomas) were collected the *SERPINA3* mRNA expression data. However, after the exclusion of cases with incomplete survival information, the remaining cases (CAMS: 267 patients; CGGA:749 patients) were involved in the statistics. Baseline information of these patients could be found in Table 1.

**Table 1:** Baseline information of patients with glioma in CAMS and CGGA

Variables	CAMS				CGGA			
	Total	*SERPINA3* ^High^	*SERPINA3* ^Low^	*p* value	Total	*SERPINA3* ^High^	*SERPINA3* ^Low^	*p* value
Gender								
Male	149	23 (15.4%)	126 (84.6%)	.296	442	244 (55.2%)	198 (44.8%)	.059
Female	118	24 (20.3%)	94 (79.7%)		307	148 (48.2%)	159 (51.8%)	
Age (years)								
≤50	125	13 (10.4%)	112 (89.6%)	.004*	542	250 (46.1%)	292 (53.9%)	<.001*
>50	142	34 (23.9%)	108 (76.1%)		207	142 (68.6%)	65 (31.4%)	
Radiotherapy								
Yes	157	21 (13.4%)	136 (86.6%)	.331	625	328 (52.5%)	297 (47.5%)	.86
No	90	17 (18.9%)	73 (81.1%)		124	64 (51.6%)	60 (48.4%)	
NA	20	9 (45.0%)	11 (55.0%)					
Chemotherapy								
Yes	202	31 (15.3%)	171 (84.7%)	.319	520	292 (56.2%)	228 (43.8%)	.002*
No	54	12 (22.2%)	42 (77.8%)		229	100 (43.7%)	129 (56.3%)	
NA^a^	11	4 (36.4%)	7 (63.6%)		0			
Grade^b^								
WHO II	3	0	3 (100.0%)	.047*	218	59 (27.1%)	159 (72.9%)	<.001*
WHO III	7	0	7 (100.0%)		240	109 (45.4%)	131 (54.6%)	
GBM	257	47 (18.3%)	210 (81.7%)		291	224 (77.0%)	67 (23.0%)	
Pathology								
Primary	231	36 (15.6%)	195 (84.4%)	.013*	502	236 (47.0%)	266 (53.0%)	<.001*
Secondary	0	0	0		25	20 (80.0%)	5 (20.0%)	
Recurrent	3	0	3 (100.0%)		222	136 (61.3%)	86 (38.7%)	
NA	33	11 (33.3%)	22 (66.7%)					
IDH mutation								
Mutant	55	3 (5.5%)	52 (94.5%)	.031*	410	132 (32.2%)	278 (67.8%)	<.001*
Wild type	148	21 (14.2%)	127 (85.8%)		339	260 (76.7%)	79 (23.3%)	
NA	64	23 (35.9%)	41 (64.1%)					
living status								
live	121	0	121 (100.0%)	.001*	293	88 (30.0%)	205 (70.0%)	<.001*
dead	146	47 (32.2%)	99 (67.8%)		456	304 (66.7%)	152 (33.3%)	
**Total**	267	47 (17.6%)	220 (82.40%)		749	392 (52.3%)	357 (47.7%)	

*Note*: Statistically significant difference (*p* value of <.05) and same below.

Abbreviations: CAMS, Chinese Academy of Medical Sciences; CGGA, Chinese Glioma Genome Atlas; GBM: glioblastoma; WHO, World Health Organization.

^a^ NA, not available.

^b^ According to WHO 2016 classification.

### 3.2 Upregulated expression of *SERPINA3* in glioma

**Figure 1:**
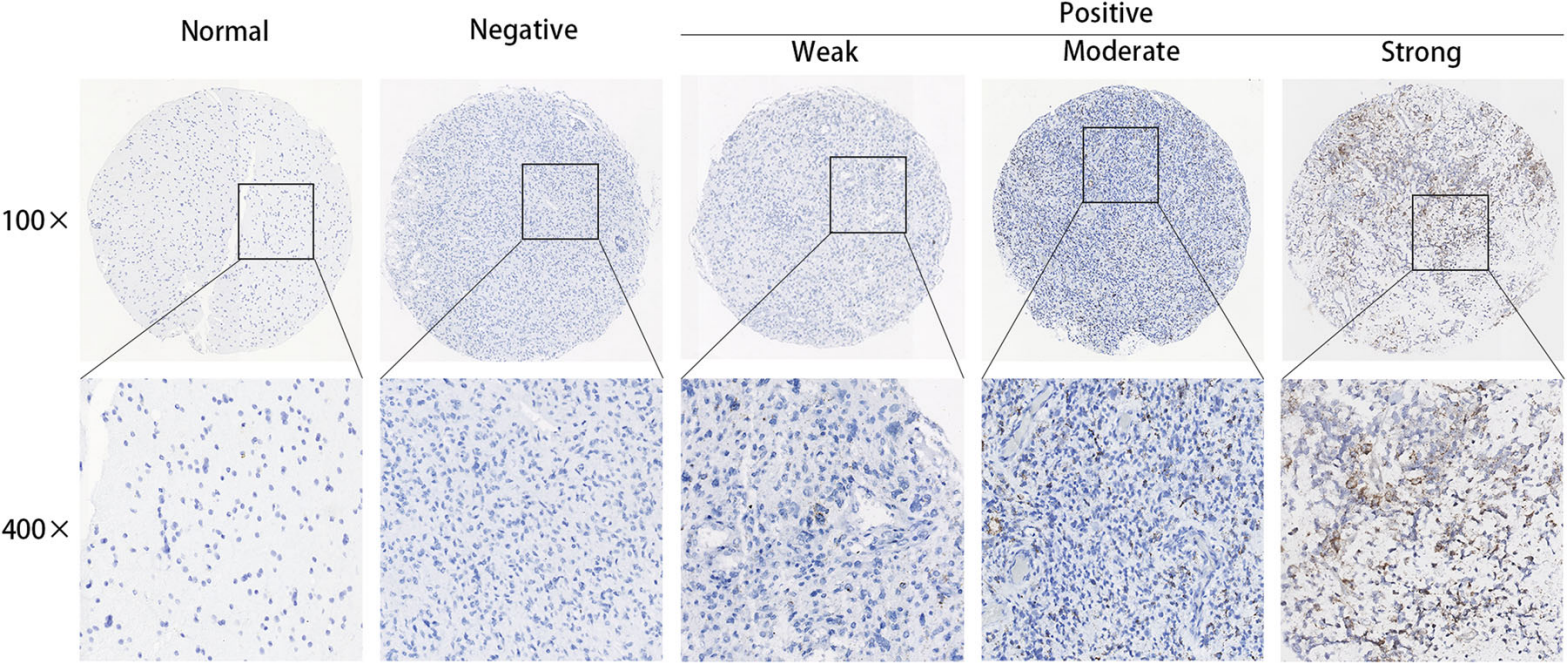
Representative RISH images of negative, low, moderate, and high *SERPINA3* mRNA expression (×100 and ×400). mRNA, messenger RNA; RISH, RNAscope in situ hybridization; SERPINA3, serpin peptidase inhibitor clade A member 3

RNAscope in situ hybridization detection of CAMS sample showed the overexpression in glioma samples. All of the gliomas had positive RISH signals for *UBC*, but none had signals for *DapB* (Figure S1). Results of RISH showed that *SERPINA3* was of high expression in 25.4% (68/267) of the tested gliomas. The positive signal is anatomically located in the cellular tumor area. Moreover, all the morphologically normal or edematous tissues presented negative *SERPINA3* mRNA expression (Figure 1).

### 3.3 *SERPINA3* mRNA level correlated with clinical characteristic

**Figure 2:**
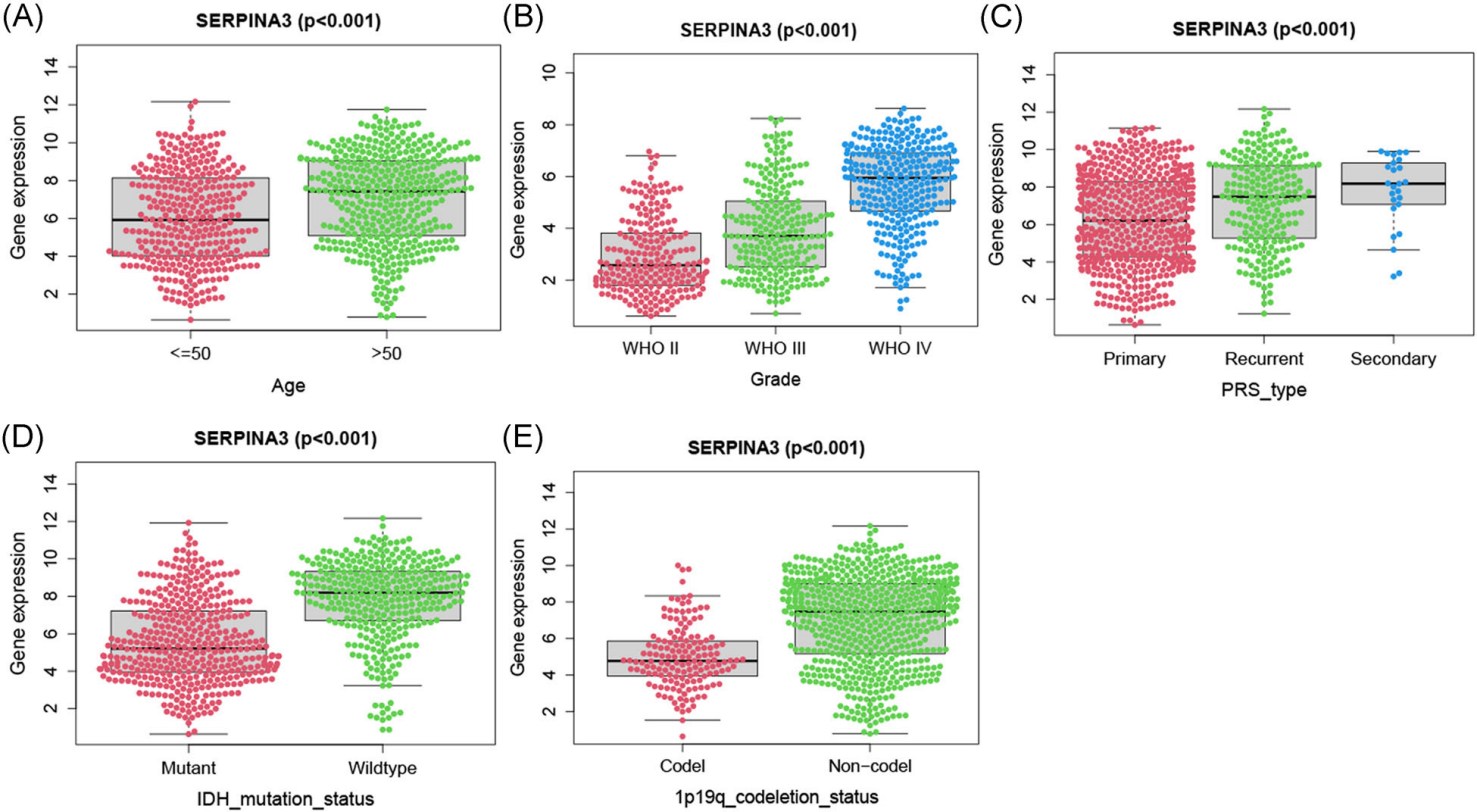
Correlation analysis between *SERPINA3* expression and clinical characteristics. Differential expression of *SERPINA3* was significantly related to (A) age (≤50, *n* = 542; >50, *n* = 207), (B) grade (WHO II, *n* = 218; WHO III, *n* = 240; WHO IV, *n* = 291), (C) PRS type (Primary, *n* = 502; Recurrent, *n* = 222; Secondary, *n* = 25). (D) *IDH* mutation status (Mutant, *n* = 410; Wild type, *n* = 339), and (E) 1p19q co‐deletion status (Codel, *n* = 155; noncodel, *n* = 594). SERPINA3, serpin peptidase inhibitor clade A member 3; WHO, World Health Organization

We examined the relationship between *SERPINA3* expression level and clinic‐pathological features of patients in CAMS and CGGA data (Table 1). The results showed that *SERPINA3* was significantly higher expressed in GBM than that in LGG (*p* = .047 and *p* < .001 in CAMS and CGGA, respectively. And same below), and in primary glioma than secondary (*p* = .013 and *p* < .001). Elderly patients (>50 years) tended to present with a higher level of *SERPINA3* mRNA expression than younger patients (≤50 years) (*p* = .004 and *p* < .001). Besides, the expression level of *SERPINA3* showed a strong correlation with *IDH1* mutation and 1p19q co‐deletion status (*p* < .001, Table 1 and Figure 2).

### 3.4 *SERPINA3* mRNA expression associated with other biomarkers of gliomas

**Figure 3:**
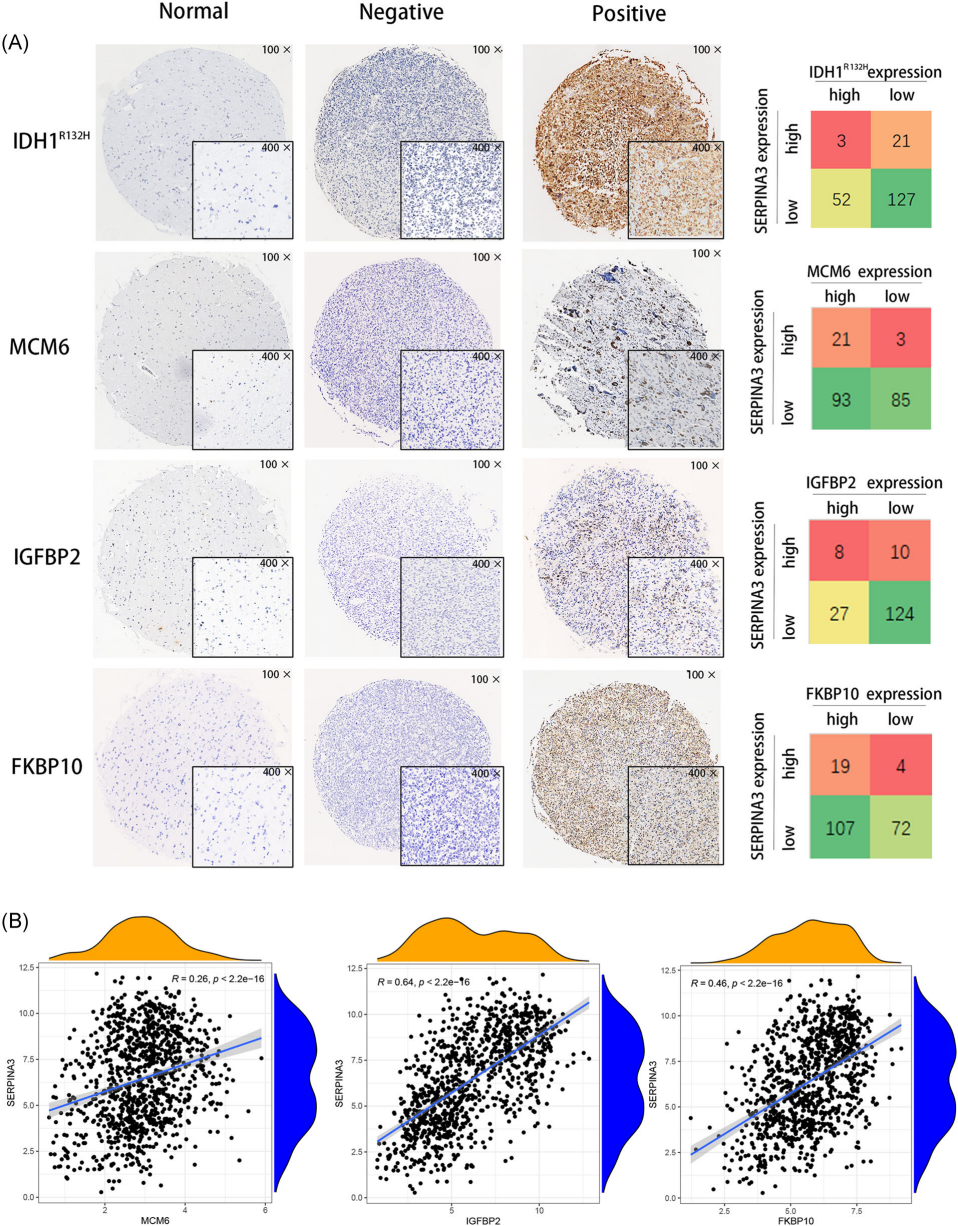
*SERPINA3* mRNA expression associated with other biomarkers. (A) Representative detection results of IDH1^R132H^, MCM6, IGFBP2, and FKBP10 and its expression relationship with SERPINA3 in CAMS gliomas. (B) Correlation of mRNA expression between *SERPINA3* and related biomarkers in CGGA dataset. Analyzed by spearman test. CGGA, Chinese Glioma Genome Atlas; FKBP10, FK506 binding protein 10; IGFBP2, insulin‐like growth factor binding protein 2; MCM6, minichromosome maintenance 6; mRNA, messenger RNA; SERPINA3, Serpin peptidase inhibitor clade A member 3

For the same cases, we previously reported high expression of *IDH1* mutation,^17^ MCM6 protein,^16^ FKBP10 protein,^18^ and *IGFBP2* mRNA.^15^ Here, we simultaneously analyzed the relationship between *SERPINA3* expression and the alterations biomarkers. We found that levels of *SERPINA3* was positively correlated with the expression of MCM6 protein, *IGFBP2* mRNA, and FKBP10 protein (*p*  = .001, .014, and .039, respectively, Table 2 and Figure 3A). Further, we examined the expression data of *SERPINA3* and these biomarkers in CGGA datasets and the transcript level of *SERPINA3* showed positively correlated with *MCM6, IGFBP2*, and *FKBP10* (Figure 3B).

**Table 2:** Relationships between *SERPINA3* and some other biomarkers in CAMS gliomas

Biomarker	Status	*SERPINA3*		*χ* ^2^ a	*p* value
		High	Low		
*IDH1*	Mutation	3	21	4.652	.031*
	Wild type	52	127		
MCM6 protein	High	21	3	9.304	.001*
	Low	93	85		
*IGFBP2* mRNA	High	8	10	6.911	.014*
	Low	27	124		
FKBP10 protein	High	19	4	3.607	.039*
	Low	107	72		

*Note*: Statistically significant difference (*p* value of <.05) and same below.

Abbreviations: CAMS, Chinese Academy of Medical Sciences; FKBP10, FK506 binding protein 10; IDH1, isocitrate dehydrogenase 1; IGFBP2, insulin‐like growth factor binding protein 2; MCM6, minichromosome maintenance 6; mRNA, messenger RNA; SERPINA3, Serpin peptidase inhibitor clade A member 3

^a^ Chi‐square test of the expression of SERPINA3 and other biomarkers.

### 3.5 *SERPINA3* serves as a prognostic biomarker in gliomas

**Figure 4:**
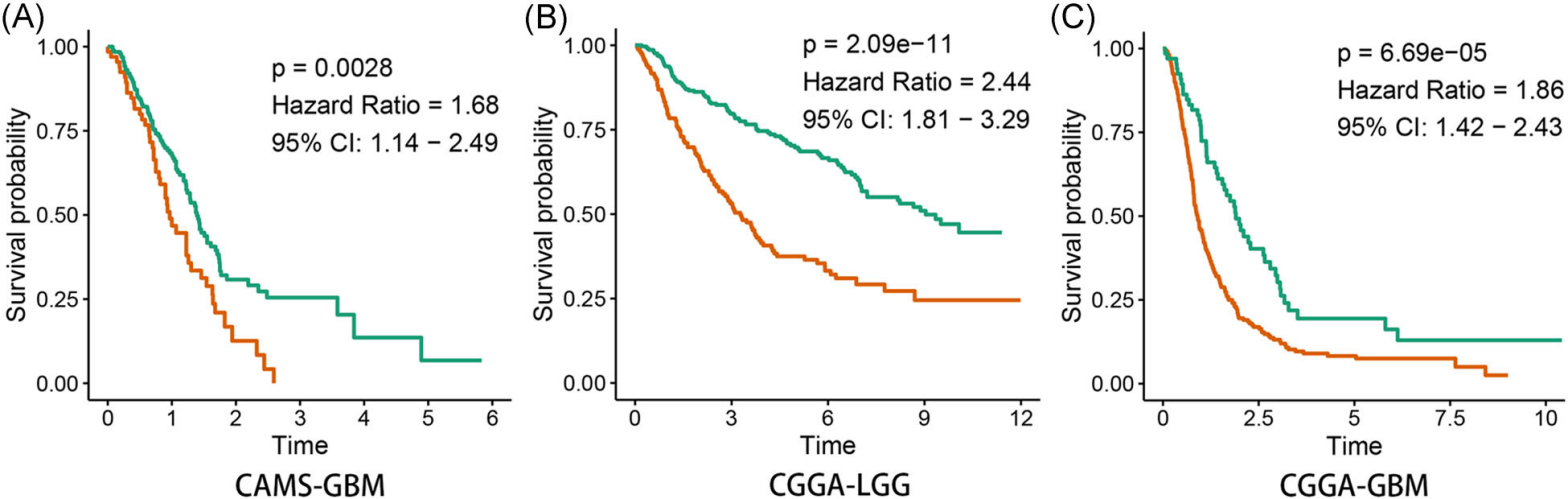
Overexpression of *SERPINA3* mRNA predicted shorter survival for patients with GBM in CAMS (A) while showed similar results in LGG and GBM in CGGA (B and C) datasets. CAMS, Chinese Academy of Medical Sciences; GBM, glioblastoma; mRNA, messenger RNA; SERPINA3, Serpin peptidase inhibitor clade A member 3

Since the 97.0% (259/267) of patients in CAMS data are GBM, we could only observed the prognosis value of *SERPINA3* in GBM. Kaplan–Meier survival analysis showed that the expression levels of *SERPINA3* were negatively related to the prognosis of patients with GBM (*n*  = 259, *p* < .001, Figure 4A). Subsequently, the prognosis value of *SERPINA3* mRNA was validated in the data of CGGA through the same methods. The results indicated that the high level of *SERPINA3* mRNA showed a strong correlation with the dismal prognosis of patients in both LGG and GBM (*n*  = 291 and 458, respectively, both *p* < .001, Figure 4B,C).

**Figure 5:**
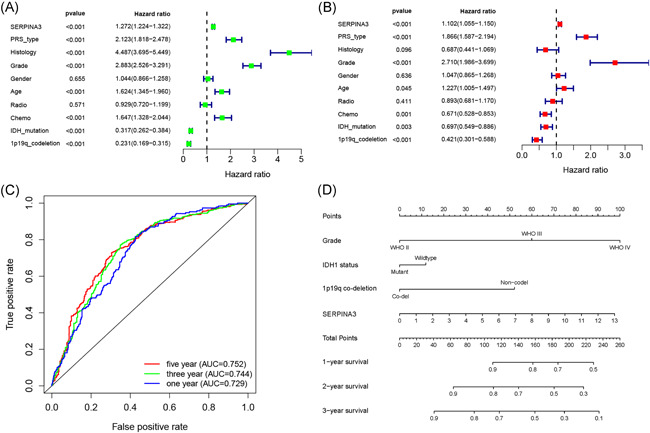
Survival prediction efficiency for *SERPINA3* in glioma using CGGA sample. (A and B) Univariate and multivariate analysis of *SERPINA3*. (C) Receiver operator characteristic curve analysis of *SERPINA3*. AUC, area under the curve. (D) Nomogram showed the prediction of survival possibilities for glioma patients in 1, 2, 3 years. CGGA, Chinese Glioma Genome Atlas; SERPINA3, Serpin peptidase inhibitor clade A member 3

Result of univariate and multivariate Cox analyse indicated that high expression of *SERPINA3* was an indepent shorter survival indicator for glioma patients (Figure 5A,B). Tine‐ROC analyses showed that AUC of *SERPINA3* expression in 1, 3, 5 years was 0.752, 0.744, 0.729, respectively (Figure 5C). Combining with clinical parameters including grade, *IDH1* mutation, and 1p19q co‐deletion status, we could predict the survival time of glioma patients more precisely. The results were presented in the nomogram (Figure 5D) and had been validated (shown in Figure S2).

### 3.6 DEGs and enrichment analysis

**Figure 6:**
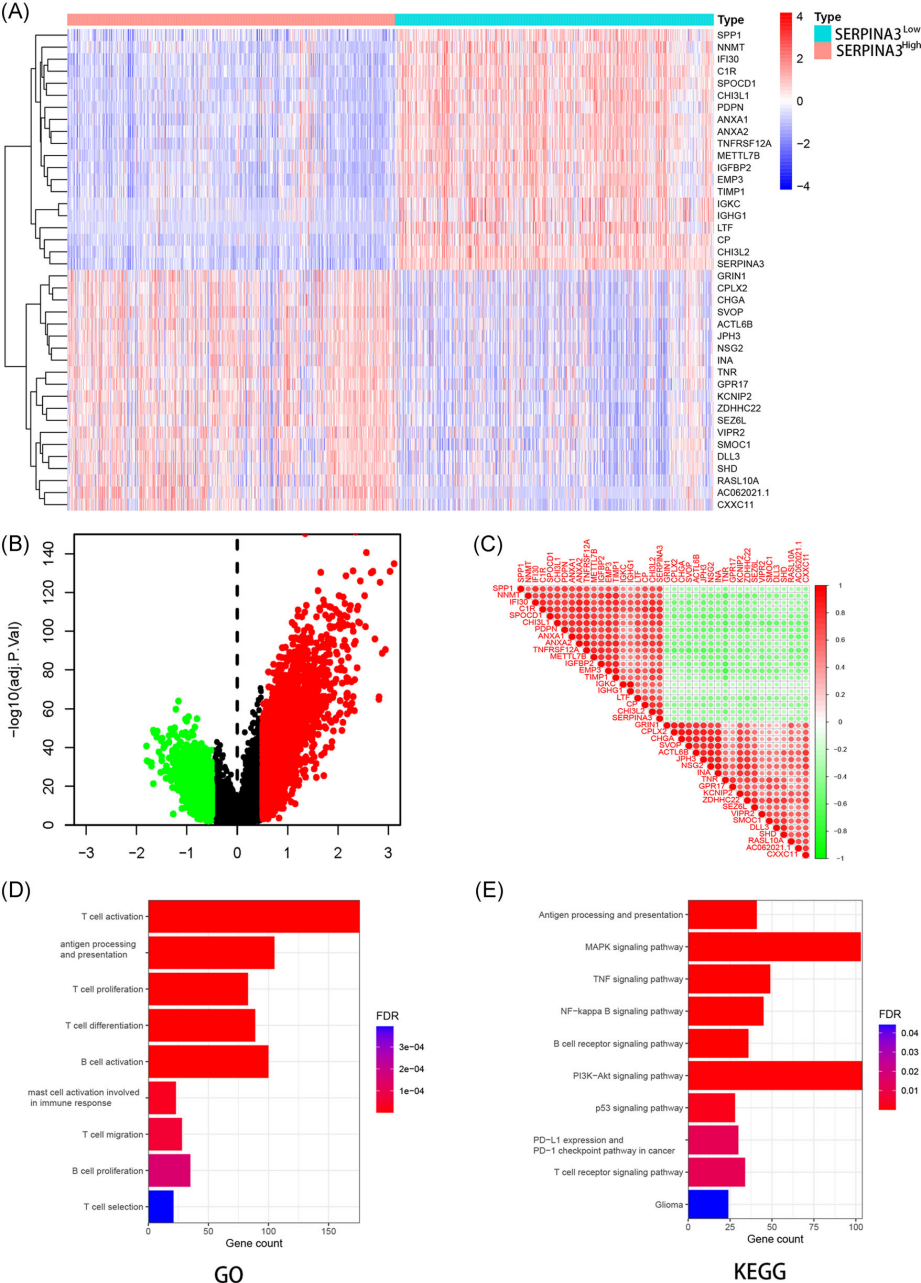
DEG and enrichment analysis for SERPINA3 in CGGA dataset. (A) Heatmap for DEGs generated by comparison of the *SERPINA3* ^high^ group vs. *SERPINA3* ^low^. Row name of headman is the gene name, and the column name is the ID of samples which not showed in a plot. (B) Volcano map of distribution of DEGs. Red point shows upregulated DEGs while green shows downregulated with SERPINA3. (C) Heatmap of correlations between differential genes. Red circles represents positively correlation, green represents negative. (D and E) GO and KEGG analysis for SERPINA3 in glioma. DEG, differentially expressed gene; GO, Gene Ontology; SERPINA3, Serpin peptidase inhibitor clade A member 3

We compared the gene expression differences between high and low *SERPINA3* expression groups in CGGA using the DESeq. 2 package. A total of 3280 genes and 829 genes were identified as upregulated and downregulated DEGs, respectively. The DEGs were exhibited in the volcano map (Figure 6B). The expression of the 20 genes with the largest differences in up‐ and downregulation in CGGA individuals was presented in the heatmap (Figure 6A). The correlation between any two of the 40 genes was shown in Figure 6C.

GO enrichment analysis showed that SERPINA3 were enrichment in various immune‐related terms including antigen processing and presentation, T and B cell activation, differentiation, and proliferation (Figure 6D). KEGG pathway analysis showed that SERPINA3 were mainly enriched in MAPK, TNF, P53, PI3K‐Akt, nuclear factor‐κB signaling pathway and PD‐L1 expression and PD‐1 checkpoint pathway in cancer (Figure 6E).

### 3.7 *SERPINA3* expression correlated with immune infiltration level and TME

**Figure 7:**
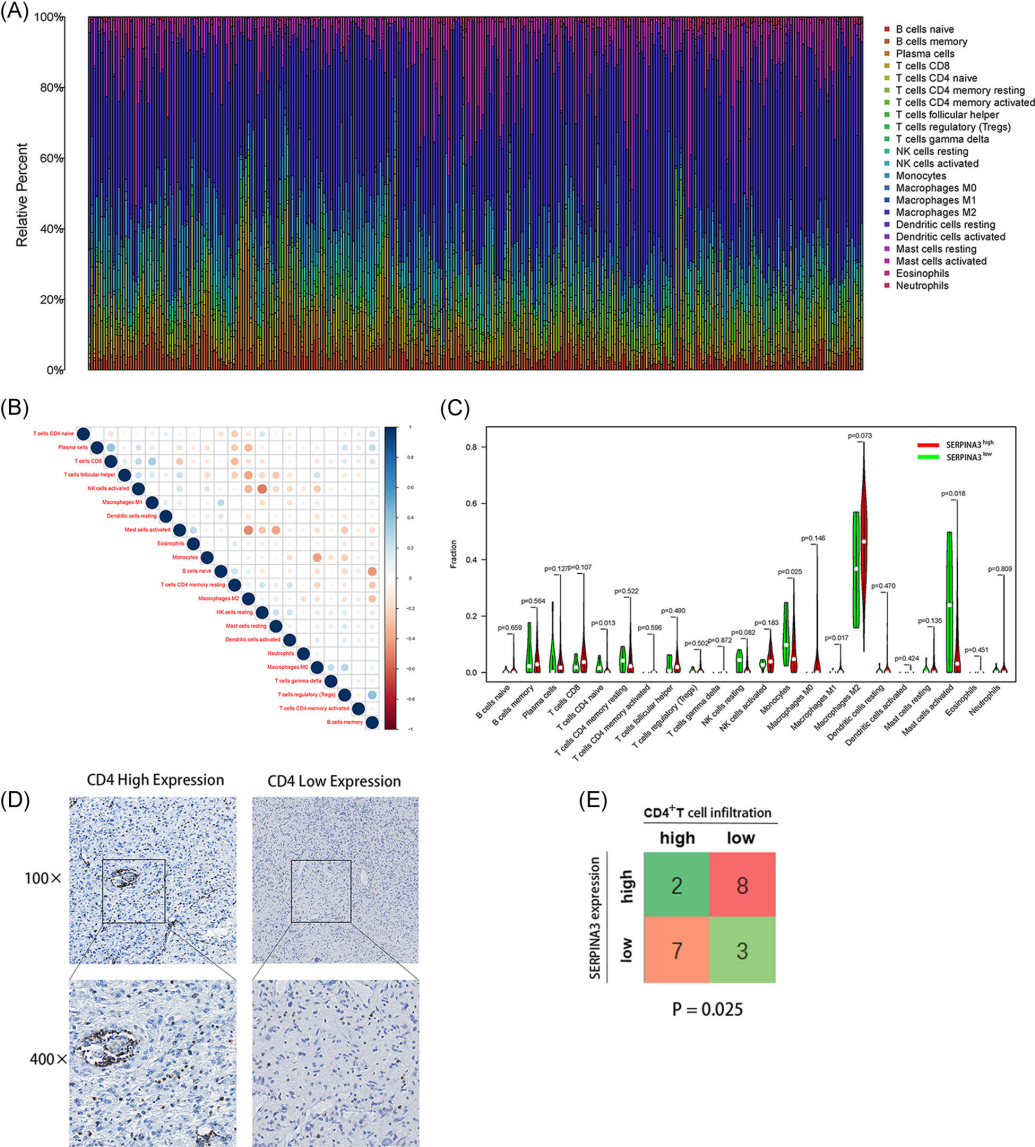
Analysis of SERPINA3 in immune‐related activities of glioma. (A) Barplot showing the proportion of 21 kinds of tumor‐infiltrating immune cells (TICs) in CGGA glioma samples. (B) Heatmap showing the correlation between 21 kinds of TICs. The shade of each tiny color box represented corresponding correlation value between two cells, and Pearson coefficient was used for the significance test. (C) Violin plot showed the ratio differences of 21 kinds of immune cells between CGGA glioma samples with low or high SERPINA3 expression. (D) Representative IHC images of high‐ and low‐ expression of CD4^+^ cells. (E) Relationship between expression of *SERPINA3* and CD4^+^ T cells infiltration in glioma. CGGA, Chinese Glioma Genome Atlas; SERPINA3, serpin peptidase inhibitor clade A member 3

We further analyzed the relationship between *SERPINA3* expression and immune cells infiltrating tumors. The comparative content of immune cells in each case was represented by a barplot (Figure 7A) and correlations among various clusters of immune cells were showed (Figure 7B). We invested the differences of in the immune cells level between subgroups *SERPINA3* ^high^ VS *SERPINA3* ^low^. The results showed that high expression of *SERPINA3* was negatively correlated with levels of M1 Macrophages, CD4 native T cell, monocytes, and Mast cell activated (*p* = .017, .013, .025, and .018, respectively, Figure 7C). Immune infiltration analysis of TCGA glioma patients in different methods showed similarty results (Table S1).

CD4^+^ T cell play a vital part in the immune procession of glioma.^19^ To further test the correlation between *SERPINA3* mRNA expression and the tumor immune infiltrating of CD4^+^ T cell in tissues, we performed the IHC experiment in 20 gliomas. The density of CD4 signal were divided into high‐ and low‐score groups separately according to the IHC staining (Figure 7D). As shown in Figure 7E, 8/10 *SERPINA3* ^high^ patients showed low CD4^+^ T cell in‐filtration while 3/10 *SERPINA3* ^low^ patients showed low CD4^+^ T cell infiltration (*p* = .025).

## 4 DISCUSSION

Gliomas are one of the most aggressive and common cancers, therefore, exploring novel molecular alterations in glioma may provide valuable insights for the design of effective therapeutic strategies. This study showed that *SERPINA3* expression is associated with glioma malignancy, including higher grade tumours and shorter patient survival time. Moreover, SERPINA3 may play a crucial role in the immune response. Specifically, SERPINA3 was found to be negatively associated with M1 Macrophages, T cell CD4 native, monocytes, and activated mast cells. Moreover, expression of *SERPINA3* correlated with low CD4^+^ T cell infiltration in glioma tissues. Overexpression of *SERPINA3* mRNA in high grade gliomas suggests that SERPINA3 may play a key role in the progression of glioma cells.

Herein, the proportion of *SERPINA3* overexpression in glioma tissues ranged from 17.6%–52.3% in different datasets. In addition to the variation between the CGGA and CAMS samples, one of the reasons for such a large difference may be due to different detection methods. In the CGGA dataset, *SERPINA3* was measured by transcriptome sequencing whereas RNAscope in situ hybridisation, which has high specificity and sensitivity, thereby providing more visible and accurate results, was used to evaluate the CAMS samples. In addition, the threshold of high expression was determined based on the prognostic analysis of various datasets, with glioma patients with high *SERPINA3* expression tending to have poor survival. In agreement with this finding, Luo et al.^13^ reported that expression of SERPINA3 was correlated with poor survival in glioma patients, as evaluated by immunohistochemistry. Recent studies have shown that SERPINA3 acts as a key molecule for survival prediction in various cancers.^11^, ^20^ The present study not only demonstrates that *SERPINA3* is a negative prognostic indicator, but also that it has a valuable prognostic performance for 1‐, 3‐, and 5‐year survival. Moreover, a survival prediction model based on *SERPINA3* mRNA expression and other factors in glioma patients was established for the first time. These analyses showed that SERPINA3 can play a significant role in predicting the survival of glioma patients.

A previous study found that SERPINA3 promotes the proliferation of melanoma and human liver cancer cells.^20^, ^21^ In the present study, the relationship between SERPINA3 and other previously verified proliferation‐related molecules, including MCM6,^16^ *IGFBP2*,^15^ and FKBP10^18^ was evaluated. A strong correlation between the expression of *SERPINA3* and these molecules in glioma suggests that SERPINA3 may participate in the proliferation of glioma cells. In addition, SERPINA3 was related to the PI3K/Akt and MAPK/Ras/Raf pathways, as indicated by the biological function analysis. These pathways have been shown to be involved in the regulation of malignant glioma cell proliferation.^22^, ^23^ Taken together, these results indicate that SERPINA3 may promote the proliferation and strengthen the malignant progression of glioma cells.

In the present study, biological analysis showed that SERPINA3 is highly enriched in multiple immune‐related terms, including activation, differentiation, and proliferation of T cells, which was in accordance with a previous study on endometrial carcinoma.^11^ Thus, it is reasonable to assume that SERPINA3 may play an essential role in the immune process of cancer. Increasing evidences have demonstrated the importance of the tumour microenvironment in cancer development, and an increasing number of studies have shown that tumour‐infiltrating immune cells can serve as promising indicators of therapeutic effects.^24^ Infiltrated inflammatory cells, tissue‐resident cells, and brain vasculature constitute the glioma microenvironment.^25^ Moreover, a reduced number of inflammatory cells in the glioma microenvironment may prevent an efficient immune surveillance.^26^ Herein, the role of SERPINA3 in the regulation of the tumour microenvironment in glioma tissues was analysed in detail for the first time. Crosstalk between microglia and peripheral macrophages in the microenvironment of glioma can block the immune response of effector T cells through the activation of immune checkpoint proteins, such as B7‐H4.^27^ Macrophage differentiation is induced and regulated by various microenvironmental signals, leading to M1, which can promote inflammatory reactions, and M2, which can regulate immunity, repair, and remodelling tissue. Given that *SERPINA3* levels were herein negatively correlated with the number of M1 macrophages, suggests that SERPINA3 may downregulate the inflammatory reaction of glioma cells.

A recent study showed that SERPINA3 is a novel target of STAT3.^21^ Activation of STAT3 is critical for tumour‐induced immune tolerance and avoidance in the glioblastoma microenvironment. For example, interleukin‐2‐mediated STAT3 activity expands tumour‐associated regulatory T cells and enhances *Foxp3* expression in CD4^+^ T cells.^28^ Based on these findings, whether SERPINA3 affects CD4^+^ T cells in glioma by affecting STAT3 should be further investigated. Alternatively, monocytes are important regulators in cancer development and contribute to antitumour immunity through phagocytosis, remodelling of the extracellular matrix, and recruitment of lymphocytes.^29^ We found that the number of monocytes in the tumour intra‐environment was reduced in the *SERPINA3* ^high^ group, which may be a possible method for the regulation of immunity. These results suggest that SERPINA3 is involved in promoting immunosuppression in the glioma microenvironment.

There is now high hope for the efficacy of various immune therapy strategies in glioma based on immune checkpoint inhibition. Anti‐PD‐L1 antibodies, such as nivolumab or pembrolizumab, have shown powerful antitumour effects. The relationship between PD‐L1 and T cell infiltration in glioma, as well as the therapeutic effects of anti‐PD‐1/PD‐L1 antibodies, remain largely elusive, which may reflect the specificity of the cellular and structural microenvironment in the brain.^30^ Multiple regulators influence PD‐L1 expression at the RNA and protein levels and drug intervention via direct regulation.^31^ The present analyses showed that high *SERPINA3* expression was positively correlated with *PD‐L1* expression (*p* < .001), suggesting that SERPINA3 may be involved in the PD‐L1‐mediated immune escape pathway. Further experiments should be performed to explore the inner mechanisms between these two molecules, and the therapeutic effect of combined inhibitory approaches should be validated.

## 5 CONCLUSION

The present study demonstrates that *SERPINA3* is overexpressed in glioma tissues and is involved in the proliferation of glioma cells. Moreover, SERPINA3 promotes immune suppression in the glioma microenvironment. Thus, SERPINA3 may serve as a novel prognostic biomarker and therapeutic target in glioma. Nonetheless, analysis of large‐scale dataset and mechanistic studies are still needed to validate the function of SERPINA3 in glioma.

## CONFLICT OF INTERESTS

The authors declare that there are no conflict of interests.

## AUTHOR CONTRIBUTIONS

Jing‐Hai Wan and Ming‐Rong Wang designed the study, analyzed the data, and revised the manuscript. Qing Yuan designed the study, performed the experiments, analyzed the data and drafted the manuscript. Hong‐Qing Cai designed the study, analyzed the data, and revised the manuscript. Song‐Quan Wang, Guang‐Tao Zhang, Zhi‐Dan Liu, and Jie He contributed materials, collected clinical information, performed the experiments and analyzed the data.

## Supporting information

Supplementary information.

Supplementary information.

Supplementary information.

## Data Availability

Data of CGGA part during the study are available in a repository or online in accordance with funder data retention policies (http://www.cgga.org.cn/). Data of CAMS part during the study are available from the corresponding author by request (wanjinghai@sina.com [J‐H.W]; and phonecy@126.com [H‐Q.C]).

## References

[1] Ostrom QT , Bauchet L , Davis FG , et al. The epidemiology of glioma in adults: a “state of the science” review. Neuro Oncol. 2014;16(7):896‐913 24842956 10.1093/neuonc/nou087 PMC4057143

[2] Stupp R , Mason WP , Van den Bent MJ , et al. Radiotherapy plus concomitant and adjuvant temozolomide for glioblastoma. The N Engl J Med. 2005;352(10):987‐996 15758009 10.1056/NEJMoa043330

[3] Wootton JC , Baron AJ , Fincham JR . Molecular‐based recursive partitioning analysis model for glioblastoma in the temozolomide era: a correlative analysis based on NRG oncology RTOG 0525. JAMA oncology. 2017;3(6):784‐792 28097324 10.1001/jamaoncol.2016.6020 PMC5464982

[4] Gilbert MR , Wang M , Aldape KD , et al. Dose‐dense temozolomide for newly diagnosed glioblastoma: a randomized phase III clinical trial, Journal of clinical oncology. Official Journal of the Am Soc Clin Oncol. 2013;31(32):4085‐9110 10.1200/JCO.2013.49.6968 PMC3816958 24101040

[5] Demyashkin GA , Nikitin PV . [IDH1‐ and IDH2‐mutations in brain glial tumors ‐ the new antioncogenic mechanism], Zhurnal nevrologii i psikhiatrii imeni S.S. Korsakova. 2018;118(4):134‐139 10.17116/jnevro201811841134-139 29863707

[6] Cairncross JG , Ueki K , Zlatescu MC , et al. Specific genetic predictors of chemotherapeutic response and survival in patients with anaplastic oligodendrogliomas J Natl Cancer Inst 1998 90(19)1473‐14739 9776413 10.1093/jnci/90.19.1473

[7] Arita H , Yamasaki K , Matsushita Y , et al. A combination of TERT promoter mutation and MGMT methylation status predicts clinically relevant subgroups of newly diagnosed glioblastomas. Acta Neuropathol Commun. 2016;4(1):79. 10.1186/s40478-016-0351-2 27503138 PMC4977715

[8] Baker C , Belbin O , Kalsheker N , Morgan K . SERPINA3 (aka alpha‐1‐antichymotrypsin), Frontiers in bioscience. A Journal And Virtual Library. 2007;12:2821‐3510 10.2741/2275 17485262

[9] Pointner H , Flegel U . Nuclear α1‐antichymotrypsin promotes chromatin condensation and inhibits proliferation of human hepatocellular carcinoma cells. Gastroenterology. 2013;144(4):818‐828 e410. 23295442 10.1053/j.gastro.2012.12.029

[10] Chelbi ST , Wilson ML , Veillard AC , et al. Genetic and epigenetic mechanisms collaborate to control SERPINA3 expression and its association with placental diseases. Hum Mol Gen. 2012;21(9):1968‐1978 22246292 10.1093/hmg/dds006

[11] Zhou ML , Chen FS , Mao H . Clinical significance and role of up‐regulation of SERPINA3 expression in endometrial cancer. World J Clin Cases. 2019;7(15):1996‐2002 31423431 10.12998/wjcc.v7.i15.1996 PMC6695533

[12] Nimbalkar VP , Kruthika BS , Sravya P , et al. Differential gene expression in peritumoral brain zone of glioblastoma: role of SERPINA3 in promoting invasion, stemness and radioresistance of glioma cells and association with poor patient prognosis and recurrence. JNO. 2021;152(1):55‐65. 10.1007/s11060-020-03685-4 33389566

[13] Luo D , Chen W , Tian Y , et al. Serpin peptidase inhibitor, clade A member 3 (SERPINA3), is overexpressed in glioma and associated with poor prognosis in glioma patients. Onco Targets Ther. 2017;10:2173‐2181 28458560 10.2147/OTT.S133022 PMC5403010

[14] Li T , Fu J , Zeng Z , et al. TIMER2.0 for analysis of tumor‐infiltrating immune cells. Nucleic Acids Res. 2020;48(W1):W509‐w514 32442275 10.1093/nar/gkaa407 PMC7319575

[15] Yuan Q , Cai H‐Q , Zhong Y , et al. Overexpression of IGFBP2 mRNA predicts poor survival in patients with glioblastoma. Biosci Rep. 2019;39(6):BSR20190045. 10.1042/bsr20190045 31138764 PMC6567677

[16] Cai H‐Q , Cheng ZJ , Zhang HP , et al. Overexpression of MCM6 predicts poor survival in patients with glioma. Hum Pathol. 2018;78:182‐187 29753008 10.1016/j.humpath.2018.04.024

[17] Cai H‐Q , Wang P‐F , Zhang H‐P , et al. Phosphorylated Hsp27 is mutually exclusive with ATRX loss and the IDH1R132H mutation and may predict better prognosis among glioblastomas without the IDH1 mutation and ATRX loss. J Clin Pathol. 2018;71(8):702‐707. 29550762 10.1136/jclinpath-2018-205000 PMC6204978

[18] Cai H‐Q , Zhang MJ , Cheng ZJ , et al. FKBP10 promotes proliferation of glioma cells via activating AKT‐CREB‐PCNA axis. J Biomed Sci. 2021;28(1):13. 10.1186/s12929-020-00705-3 33557829 PMC7871608

[19] Ayasoufi K , Pfaller CK , Evgin L , et al. Brain cancer induces systemic immunosuppression through release of non‐steroid soluble mediators. Brain. 2020;143(12):3629‐3652 33253355 10.1093/brain/awaa343 PMC7954397

[20] Ko E , Kim JS , Bae JW , Kim J , Park SG , Jung G . SERPINA3 is a key modulator of HNRNP‐K transcriptional activity against oxidative stress in HCC. Redox Biol. 2019;24:101217. 31121493 10.1016/j.redox.2019.101217 PMC6529774

[21] Kulesza DW , Ramji K , Maleszewska M , et al. Search for novel STAT3‐dependent genes reveals SERPINA3 as a new STAT3 target that regulates invasion of human melanoma cells. Lab Invest. 2019;99(11):1607‐1621. 10.1038/s41374-019-0288-8 31278347

[22] Aldape K , Zadeh G , Mansouri S , Reifenberger G , Von Deimling A . Glioblastoma: pathology, molecular mechanisms and markers. Acta Neuropathol. 2015;129(6):829‐848. 10.1007/s00401-015-1432-1 25943888

[23] Mao H , Lebrun DG , Yang J , Zhu VF , Li M . Deregulated signaling pathways in glioblastoma multiforme: molecular mechanisms and therapeutic targets. Cancer Invest. 2012;30(1):48‐56 22236189 10.3109/07357907.2011.630050 PMC3799884

[24] Gajewski TF , Schreiber H , Fu YX . Innate and adaptive immune cells in the tumor microenvironment. Nature Immunol. 2013;14(10):1014‐1022 24048123 10.1038/ni.2703 PMC4118725

[25] Locarno CV , Simonelli M , Carenza C , et al. Role of myeloid cells in the immunosuppressive microenvironment in gliomas. Immunobiology. 2020;225(1):15185310.1016/j.imbio.2019.10.002 10.1016/j.imbio.2019.10.002 31703822

[26] Price G , Bouras A , Hambardzumyan D , Hadjipanayis CG . Current knowledge on the immune microenvironment and emerging immunotherapies in diffuse midline glioma. EBioMedicine. 2021;69:103453. 10.1016/j.ebiom.2021.103453 34157482 PMC8220552

[27] Yao Y , Ye H , Qi Z , et al. B7‐H4(B7x)‐mediated cross‐talk between glioma‐initiating cells and macrophages via the IL6/JAK/STAT3 pathway lead to poor prognosis in glioma patients. Clin Cancer Res. 2016;22(11):2778‐2790 27001312 10.1158/1078-0432.CCR-15-0858 PMC4891287

[28] Kortylewski M , Kujawski M , Wang T , et al. Inhibiting Stat3 signaling in the hematopoietic system elicits multicomponent antitumor immunity. Nature Med. 2005;11(12):1314‐1321 16288283 10.1038/nm1325

[29] Zhang N , Dai Z , Wu W , et al. The predictive value of monocytes in immune microenvironment and prognosis of glioma patients based on machine learning. Front Immunol. 2021;12:656541 33959130 10.3389/fimmu.2021.656541 PMC8095378

[30] Huang J , Liu F , Liu Z , et al. Immune Checkpoint in Glioblastoma: Promising and Challenging. Front Pharmacol. 2017;8:24210 10.3389/fphar.2017.00242 PMC5422441 28536525

[31] Zhang H , Dai Z , Wu W , et al. Regulatory mechanisms of immune checkpoints PD‐L1 and CTLA‐4 in cancer. J Exp Clin Cancer Res. 2021;40(1):184. 10.1186/s13046-021-01987-7 34088360 PMC8178863

