# Supplementary figures and images for: Highly expressed of SERPINA3 indicated poor prognosis and involved in immune suppression in glioma
Source: Immun Inflamm Dis. 2021 Aug 27;9(4):1618–30. doi: 10.1002/iid3.515 (PMC8589354; doi:10.1002/iid3.515)

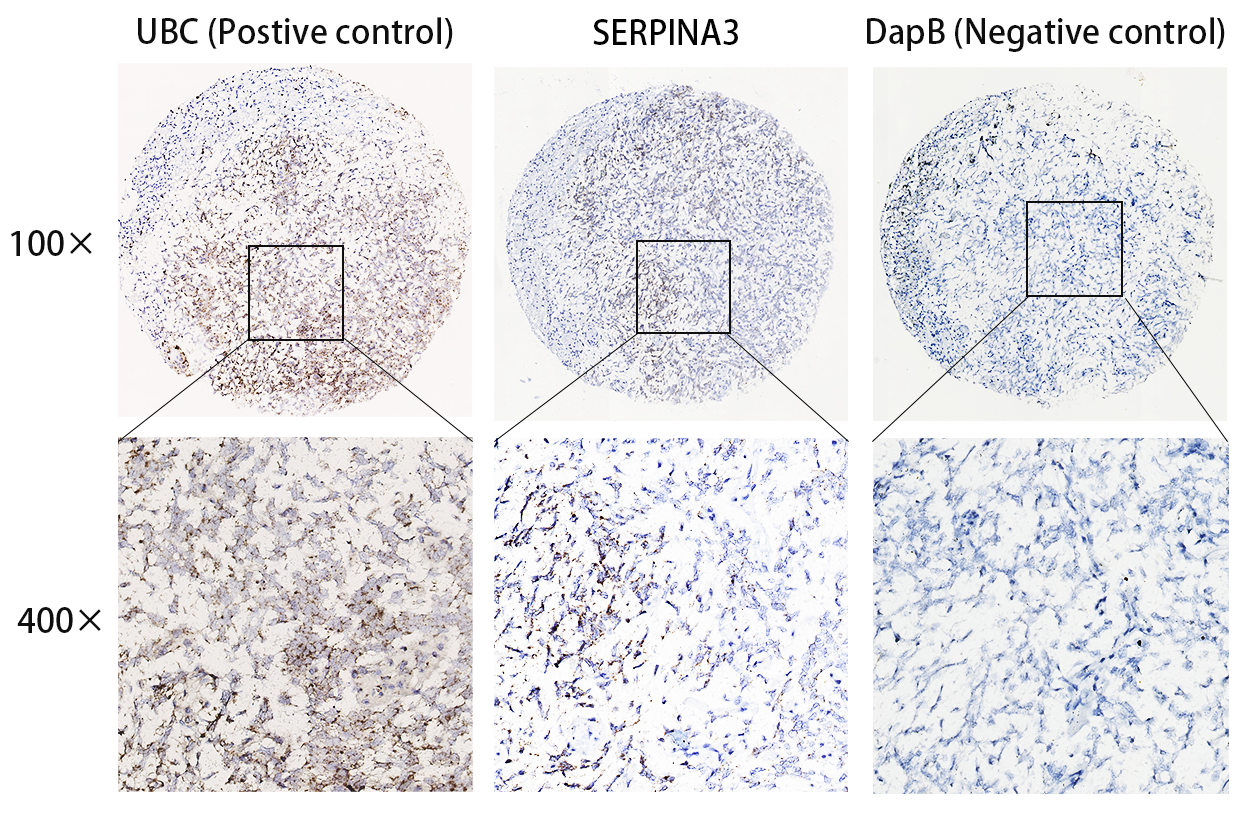

Supplement: Supplementary file 1 — Supplementary information. [file IID3-9-1618-s001.tif]

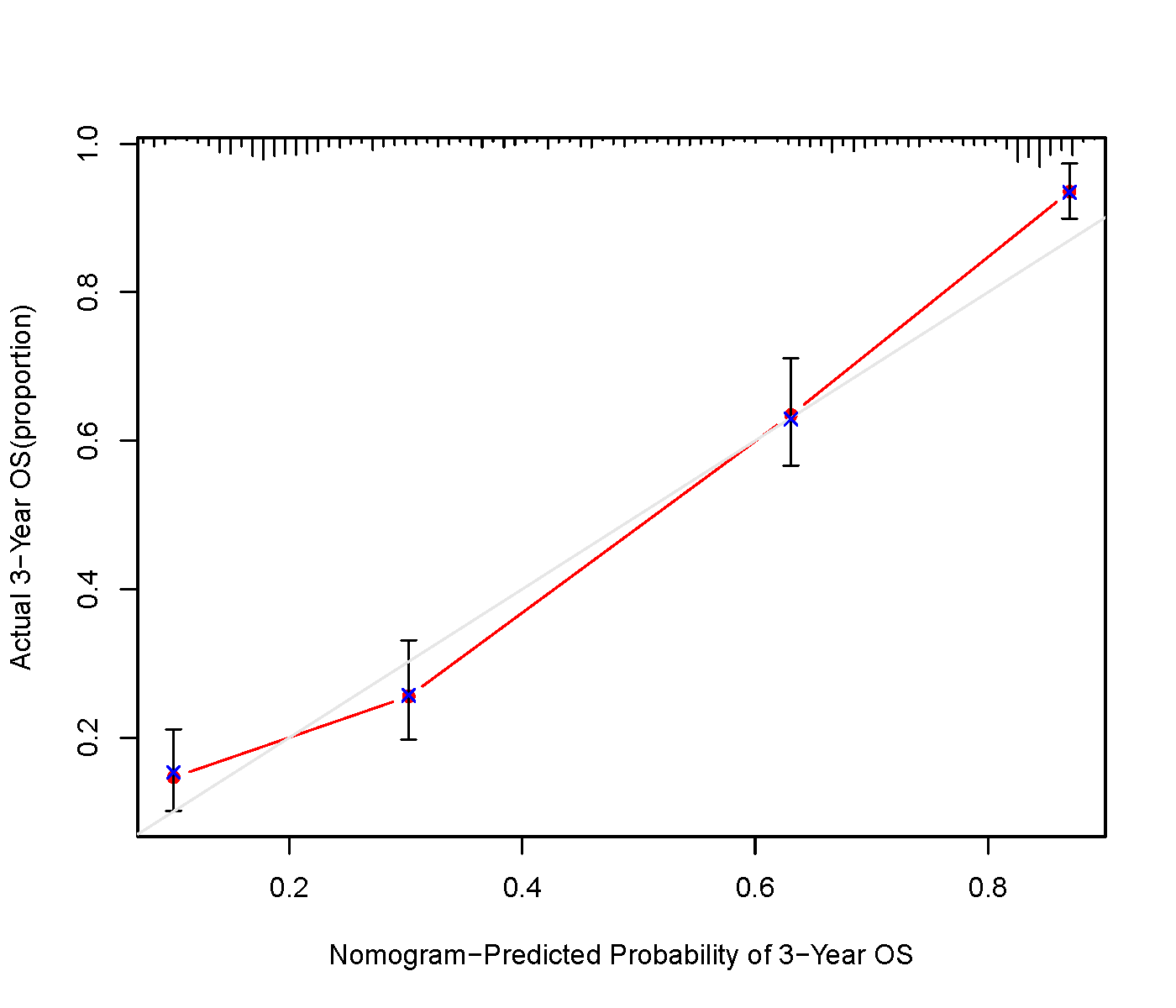

Supplement: Supplementary file 2 — Supplementary information. [file IID3-9-1618-s003.tif]
