# Supplementary material for: Highly expressed of SERPINA3 indicated poor prognosis and involved in immune suppression in glioma
Source: Immun Inflamm Dis. 2021 Aug 27;9(4):1618–30. doi: 10.1002/iid3.515 (PMC8589354; doi:10.1002/iid3.515)
Supplement: Supplementary file 3 — Supplementary information. [file IID3-9-1618-s002.docx]

Supplmentry Table [1](#MEP_L_tbl1): Immune inflammation analysis of *SERPINA3* expression in TCGA glioma patients (LGG▒+▒HGG).

| Methods^a^ | Immune Cell Type | COR^b^ | P-VAL |
| --- | --- | --- | --- |
| TIMER |  |  |  |
|  | B cell | -0.060 | 1.30E-39 |
|  | T cell CD4▒+▒ | 0.143 | 1.19E-38 |
|  | T cell CD8▒+▒ | 0.283 | 1.04E-36 |
|  | Neutrophil | 0.296 | 2.13E-39 |
|  | Macrophage | 0.250 | 7.28E-42 |
|  | Myeloid dendritic cell | 0.235 | 9.74E-22 |
| CIBERSORT |  |  |  |
|  | T cell CD4▒+▒naive | -0.199 | 3.14E-45 |
|  | T cell CD4▒+▒memory resting | 0.153 | 2.54E-41 |
|  | T cell CD4▒+▒memory activated | 0.053 | 1.16E-45 |
|  | T cell follicular helper | -0.096 | 1.08E-43 |
|  | T cell regulatory (Tregs) | 0.234 | 1.94E-45 |
|  | T cell gamma delta | 0.0275 | 1.15E-45 |
|  | Monocyte | -0.138 | 1.16E-36 |
|  | Macrophage M1 | -0.068 | 5.91E-45 |
|  | Mast cell activated | -0.064 | 2.66E-44 |
| CIBERSORT-ABS |  |  |  |
|  | T cell CD4▒+▒naive | -0.191 | 1.25E-45 |
|  | Monocyte | 0.195 | 2.28E-44 |
|  | Macrophage M1 | 0.124 | 1.55E-45 |
|  | Mast cell activated | 0.072 | 1.63E-45 |
| QUANTI-SEQ |  |  |  |
|  | Macrophage M1 | 0.364 | 3.35E-45 |
|  | T cell CD4▒+▒(non-regulatory) | 0.019 | 3.34E-45 |
|  | Monocyte | -0.049 | 1.81E-223 |
|  | Macrophage/Monocyte | -0.049 | 1.81E-223 |
| XCELL |  |  |  |
|  | T cell CD4▒+▒naive | -0.059 | 1.19E-45 |
|  | Macrophage M1 | -0.413 | 1.47E-44 |
|  | Mast cell | -0.042 | 3.46E-45 |
| EPIC |  |  |  |
|  | T cell CD4▒+▒ | -0.204 | 2.17E-42 |
|  | T cell CD8▒+▒ | -0.118 | 1.31E-44 |
|  | Macrophage | 0.324 | 2.45E-44 |

Note: a, different inflammation analysis methods (See details in reference#[17](#MEP_L_bib17)); b, correlation coefficient of T-test for the immune cells and cotain of SERPINA3.

**Supplmentry** Figure [1](#MEP_L_fig1)**.**

Representative RISH images of SERPINA3 mRNA expression, negative control (DapB) and positive control (UBC)

**Supplmentry** Figure [2](#MEP_L_fig2)**.**

Nomogram-predicted probability of 3-year OS in glioma patients.
